# Differential value of diffusion kurtosis imaging and intravoxel incoherent motion in benign and malignant solitary pulmonary lesions

**DOI:** 10.3389/fonc.2022.1075072

**Published:** 2023-01-12

**Authors:** Lu Xiang, Hong Yang, Yu Qin, Yun Wen, Xue Liu, Wen-Bing Zeng

**Affiliations:** ^1^ Department of Radiology, Chongqing University Three Gorges Hospital, Chongqing, China; ^2^ PET-CT Center, Chongqing University Three Gorges Hospital, Chongqing, China; ^3^ College of Medical Imaging, North Sichuan Medical College, Sichuan, China; ^4^ Chongqing University School of Medicine, Chongqing, China

**Keywords:** solitary pulmonary lesion, lung cancer, magnetic resonance imaging, diffusion kurtosis imaging, intravoxel incoherent motion, histogram analysis

## Abstract

**Objective:**

To investigate the diagnostic value of diffusion kurtosis imaging (DKI) and intravoxel incoherent motion (IVIM) whole-lesion histogram parameters in differentiating benign and malignant solitary pulmonary lesions (SPLs).

**Materials and Methods:**

Patients with SPLs detected by chest CT examination and with further routine MRI, DKI and IVIM-DWI functional sequence scanning data were recruited. According to the pathological results, SPLs were divided into a benign group and a malignant group. Independent samples t tests (normal distribution) or Mann‒Whitney U tests (nonnormal distribution) were used to compare the differences in DKI (Dk, K), IVIM (D, D*, f) and ADC whole-lesion histogram parameters between the benign and malignant SPL groups. The receiver operating characteristic (ROC) curve was used to evaluate the diagnostic efficiency of the histogram parameters and determine the optimal threshold. The area under the curve (AUC) of each histogram parameter was compared by the DeLong method. Spearman rank correlation was used to analyze the correlation between histogram parameters and malignant SPLs.

**Results:**

Most of the histogram parameters for diffusion-related values (Dk, D, ADC) of malignant SPLs were significantly lower than those of benign SPLs, while most of the histogram parameters for the K value of malignant SPLs were significantly higher than those of benign SPLs. DKI (Dk, K), IVIM (D) and ADC were effective in differentiating benign and malignant SPLs and combined with multiple parameters of the whole-lesion histogram for the D value, had the highest diagnostic efficiency, with an AUC of 0.967, a sensitivity of 90.00% and a specificity of 94.03%. Most of the histogram parameters for the Dk, D and ADC values were negatively correlated with malignant SPLs, while most of the histogram parameters for the K value were positively correlated with malignant SPLs.

**Conclusions:**

DKI (Dk, K) and IVIM (D) whole-lesion histogram parameters can noninvasively distinguish benign and malignant SPLs, and the diagnostic performance is better than that of DWI. Moreover, they can provide additional information on SPL microstructure, which has important significance for guiding clinical individualized precision diagnosis and treatment and has potential clinical application value.

## 1 Introduction

Lung cancer is one of the most common malignant tumors in the world and the main cause of cancer-related death (1), which seriously threatens human life and health. Solitary pulmonary lesions (SPLs) include lung nodules and lung masses. Early identification of benign and malignant SPLs is conducive to the choice of treatment for patients and is crucial to the survival rate, quality of life and prognosis of patients. At present, the identification is mainly based on imaging examinations (2). Computerized tomography (CT) is the main imaging examination method for screening lung lesions, and it is an effective and convenient method for most lesions (3). It is based on morphology and enhancement features, but it is difficult to differentiate diagnoses when the CT features are not typical. In addition, CT scan will increase unnecessary radiation exposure. With the continuous upgrading of magnetic resonance imaging (MRI) hardware and software and the development of various rapid imaging technologies (such as free breathing acquisition technology (4)), MRI has been gradually applied to the diagnosis of lung diseases. It can noninvasively evaluate benign and malignant lung diseases and aid in pathological classification, grading, TNM stage, efficacy, and prognosis of lung cancer (5). A variety of MR functional imaging technologies, such as dynamic contrast-enhanced (DCE) (6), diffusion weighted imaging (DWI) (7), intravoxel incoherent motion (IVIM) (8) and diffusion kurtosis imaging (DKI) (9), can not only show the morphological characteristics of lung lesions but also obtain functional information, such as internal diffusion and perfusion of lung lesions, to provide important information on tissue function, physiology, pathology and molecules, which has important significance for guiding clinical individualized precision diagnosis and treatment.

Histogram analysis is a newly developed method based on different voxel values of lesions, which can be applied to various signal changes. It can also provide more quantitative information, such as standard deviation, percentile, energy, entropy, skewness, and kurtosis values (10), which can be used as a potential noninvasive method for tumor diagnosis, pathological classification, grading, staging, and evaluation of efficacy and prognosis (11). Moreover, it does not require additional hardware or sequences and has high repeatability and consistency (12). DKI was first proposed by Jensen et al. (13) in 2005 and applied to brain tissue, which is very sensitive to tissue microstructure. It is conducive to the early detection of lesions and can reflect the tissue microenvironment structure more accurately, so it is widely used in the diagnosis and treatment of tumors (14). The IVIM bi-exponential model is an advanced noninvasive functional imaging technology (15). It can quantitatively provide intralesional perfusion and diffusion information by functional calculations after multiple b-value scanning without contrast agent injection, which compensates for the shortcomings of the traditional DWI mono-exponential model. DKI and IVIM combined with histogram analysis have been applied to other parts of the body (14, 16), but little research has been done on the lungs. Zheng et al. (17) showed that DWI (ADC), IVIM (D) and DKI (Kapp) could effectively distinguish benign and malignant lung lesions, and the diagnostic efficiency of the combination of the above parameters was higher than that of a single parameter. However, there was no significant difference in IVIM (D*, f) and DKI (Dapp) between the two groups. The results of Wan et al. (18) were not completely consistent with those of Zheng et al. (17), and they believed that the DWI (ADC), IVIM (D) and DKI (Dapp) values of malignant SPLs were significantly lower than those of benign SPLs, but IVIM (D*, f) and DKI (Kapp) could not effectively distinguish benign and malignant SPLs. The above studies indicate that IVIM and DKI have certain value in the differential diagnosis of benign and malignant lung lesions, but they are still in the exploratory stage. In addition, these studies (17, 18) used quantitative parameters describing ROI at a single level to assess lung lesions, rarely combined with whole lesion histogram methods for further analysis, and did not reflect the overall characteristics of lesions.

Therefore, on the basis of previous studies, this study intends to delineate the ROI of the whole lesion layer by layer to evaluate its characteristics more comprehensively, objectively and accurately, thus obtaining more complete biological information of the lesion. Our objective was to explore the value of DKI and IVIM histogram parameters in differentiating benign and malignant SPL by whole-lesion histogram analysis, and to compare them with mono-exponential DWI histogram parameters.

## 2 Materials and methods

### 2.1 Research objects

The Medical Ethics Committee of our hospital approved the prospective study protocol. From May 2021 to November 2022, a total of 152 patients with SPLs detected by chest CT examination and further routine MRI, DKI and IVIM-DWI functional sequence scanning were recruited. The following inclusion criteria were used: (1) solid or partially solid SPL (solid component > 50%) with a diameter > 10 mm; (2) no recent history of acute inflammation; and (3) MRI scan performed within 1 week after lung lesions were detected by CT examination, with no radiotherapy, chemotherapy or other treatment performed before the scan. The following exclusion criteria were used: (1) MRI contraindications, such as claustrophobia, foreign metal bodies in the body, and inability to complete the examination; (2) parameter values not measured due to heavy motion artifacts or magnetic sensitivity artifacts; and (3) no pathological results. Pathological findings were obtained by surgical resection, CT-guided percutaneous biopsy or fiberoptic bronchoscopic biopsy. All patients signed informed consent for examination.

### 2.2 MRI examination method

All participants were scanned with a 3.0T MRI imaging device (MAGNETOM Vida, Siemens Healthcare, Germany) and a 16-channel phased front coil. The patient was scanned in the supine position while breathing calmly and uniformly, and a STAR-VIBE sequence scan was performed. The coronal T2WI parameters were as follows: TR, 1400 ms; TE, 85 ms; FOV, 400 mm×100 mm; matrix, 384×80; layer thickness, 4 mm. The following parameters for axial T2WI with fat suppression techniques were used: TR, 4000 ms; TE, 95 ms; FOV, 400 mm×100 mm; matrix, 320×100; layer thickness, 4 mm. The axial T1WI parameters were as follows: TR, 2.41 ms; TE, 1.28 ms; FOV, 380 mm×100 mm; matrix, 320×50; layer thickness, 4 mm. The following parameters for single-excitation plane echo (SS-EPE) sequence DWI of axial multiple b-value breath-triggered lipid compression were used: TR, 7000 ms; TE, 58 ms; FOV, 380 mm×80 mm; matrix, 120×100; layer thickness, 4 mm; b value range, 0 to 2000 s/mm^2^ (b =0, 20, 50, 100, 150, 200, 300, 500, 800, 1000, 1200, 1600, 2000 s/mm^2^) applied simultaneously in X, Y and Z axes and applied with diffusivity sensitive gradient pulses. The total scanning time was 26 minutes and 1 second, which was tolerated by the average patient and cooperated well. Based on previous studies (19), the selection of the b value signal distribution and number in this study is a trade-off between acquisition time and SNR in the model.

### 2.3 Postprocessing of MRI images

DKI quantitative parameters are as follows: Dk, diffusion coefficient, which represents the diffusion coefficient corrected by non-Gaussian diffusion motion, and K, diffusion kurtosis coefficient, which represents non-Gaussian diffusion motion and reflects the heterogeneity and complexity of tissue microstructure (20). IVIM quantitative parameters are as follows: D, slow diffusion coefficient (ADC slow), which represents the diffusion motion of pure water molecules; D*, fast diffusion coefficient (ADC fast), which is the false diffusion coefficient generated by blood circulation, represents the incoherent movement of microcirculation in voxels and is proportional to the average capillary length and the average blood flow velocity; and f, perfusion fraction, representing the volume ratio of the microcirculation perfusion-related diffusion effect in voxels to the total diffusion effect, expressed as a percentage, which increases with the increase of tissue perfusion volume. The traditional ADC value includes the diffusion effect of water molecules and the perfusion effect of microcirculation, and the D value without the perfusion factor can more accurately reflect the diffusion conditions inside the tumor tissue (17). Dk and D are proportional to ADC.

The original images of all patients were exported from the Siemens workstation in DICOM format, and the Body MRStation software provided by Siemens was used for postprocessing analysis. The DKI model was selected for functional calculation, and pseudocolor images of Dk and K were obtained. The whole-lesion ROI was manually delineated layer by layer by reference to MRI-enhanced images. The delineated ROI was fused into a volume of interest (VOI) and then copied to other functional imaging images. The corresponding histogram parameters were obtained: mean, 10th percentile, 90th percentile, inter-quartile range (IQR), maximum (max), median, minimum (min), range, energy, entropy, kurtosis, skewness, uniformity, and variance. The VOI was copied to the IVIM and DWI models with the same software and the same method to obtain the whole-lesion histogram parameters of D, D*, f and ADC. In this study, the calculation of the ADC value was taken from the DWI data of multiple b values in the same group. Compared with the previous two b values, the ADC value calculated by multiple b values is more accurate and dependable (21). All ROI delineation avoided visible liquefaction necrosis, cavities, large blood vessels and the marginal area of the lesion because these areas have no tumor cell activity, which will reduce the difference between benign and malignant SPLs and affect the analysis results. The images of all patients were processed, and parameters were extracted separately by two thoracic radiologists blinded to the clinical and pathological data of the patients.

### 2.4 Statistical analysis

The intraclass correlation coefficient (ICC) was used to evaluate interobserver agreement for parameter measurements (0.00-0.20, poor agreement; 0.21-0.40, fair agreement; 0.41-0.60, moderate agreement; 0.61-0.80, good agreement; and 0.81-1.00, excellent agreement) (14). The Shapiro‒Wilk test was used to test the normality of the measurement data. Measurement data with a normal distribution were statistically described by the mean ± standard deviation, and comparisons between groups were performed by independent samples t tests. Measurement data that did not conform to a normal distribution were statistically described by the median (lower quartile, upper quartile) [M (P25, P75)], and comparisons between groups were performed by the Mann‒Whitney U test. Enumeration data were described by the number of cases (percentage), and comparisons between groups were performed by the χ^2^ test or Fisher’s exact probability method. The ROC and AUC were used to evaluate the diagnostic efficacy of the whole-lesion histogram parameters in differentiating benign and malignant SPLs and to determine the optimal diagnostic threshold. The DeLong method was used to compare the differences between the AUC of each parameter. Spearman rank correlation analysis was used to evaluate the correlation between the whole-lesion histogram parameters and malignant SPLs. |*r_s_* |<0.4 indicates a low correlation, 0.4<|*r_s_* |<0.7 indicates a moderate correlation, and |*r_s_* |>0.7 indicates a high correlation; *r_s_* >0 indicates a positive correlation, while *r_s_* <0 indicates a negative correlation. SPSS 25.0, MedCalc 19.4, and GraphPad Prism 9.0 software were used for statistical analysis and graph drawing. P < 0.05 was considered statistically significant.

## 3 Results

### 3.1 Clinical and imaging data

A total of 152 patients were recruited for this study, and 15 patients were excluded. The reasons for exclusion were inability to measure parameter values due to heavy motion artifacts or magnetic sensitivity artifacts (n=9) and no pathological results (n=6). A total of 137 SPL patients were included, including 86 males and 51 females aged 18-79 years, and 73 patients had a history of smoking. There were 70 cases of malignant SPLs, including adenocarcinoma (n=45), squamous cell carcinoma (n=17), large cell lung cancer (n=1), large cell neuroendocrine carcinoma (n=1), malignant melanoma (n=1), small cell lung cancer (n=4) and metastatic tumor (n=1). There were 67 cases of benign SPLs, including chronic granulomatous inflammation (n=27), pulmonary tuberculoma (n=23), organizing pneumonia (n=12), pulmonary abscess (n=2), hamartoma (n=1) and pulmonary sclerosing pneumocytoma(n=2). The clinical and imaging data are analyzed in **Table 1**. There were statistically significant differences in age and symptoms between benign and malignant SPLs. The age threshold was 55 years, the AUC was 0.637, the sensitivity was 75.71%, and the specificity was 50.75%. There were no significant differences in sex, smoking history or tumor location. The diameter of SPLs ranged from 10 to 46mm, and the volume of ROI ranged from 1.2 to 56 cm^3^. There was no significant difference in diameter and volume between benign and malignant SPLs.

**Table 1:** Comparison of clinical and imaging data between benign and malignant solitary pulmonary lesions.

Parameters	Benign (n=67)	Malignant (n=70)	*P*
Age (y)	55 (53∼64)	63.5 (55.75∼68)	0.005
Sex (male)	41 (61.19%)	45 (64.29%)	0.708
Smoking history	34 (50.75%)	39 (55.71%)	0.560
Symptom	Asymptomatic 22 (32.84%) Cough 25 (37.31%) Cough and expectoration 13 (19.40%) Blood in the sputum 6 (8.96%) Hemoptysis 6 (8.96%) Chest pain 9 (13.43%)	Asymptomatic 17 (24.29%) Cough 8 (11.43%) Cough and expectoration 33 (47.14%) Blood in the sputum 10 (14.29%) Hemoptysis 7 (10.00%) Chest pain 23 (32.86%)	P<0.001
SPL position	RUL 26 (38.81%) RML 6 (8.96%) RLL 12 (17.91%) LUL 15 (22.39%) LLL 8 (11.94%)	RUL 21 (30.00%) RML 5 (7.14%) RLL 10 (14.29%) LUL 22 (31.43%) LLL 12 (17.14%)	0.581
Pathology type	Chronic granulomatous inflammation 27 (40.30%) Pulmonary tuberculoma 23 (34.33%) Organizing pneumonia 12 (17.91%) Pulmonary abscess 2 (2.99%) Hamartoma 1 (1.49%) Pulmonary sclerosing pneumocytoma 2 (2.99%)	Adenocarcinoma 45 (64.29%) Squamous cell carcinoma 17 (24.29%) Large cell lung cancer 1 (1.43%) Large cell neuroendocrine carcinoma 1 (1.43%) Malignant melanoma 1 (1.43%) Small cell lung cancer 4 (5.71%) Metastases 1 (1.43%)	–

Enumeration data were expressed as N (%); two independent sample t-test were expressed as mean ± standard deviation; Mann-Whitney U test were expressed as M (P25,P75); RUL, right upper lobe; RML, right middle lobe; RLL, right lower lobe; LUL, left upper lobe; LLL, left lower lobe.

### 3.2 Whole-lesion histogram parameters

**Figure 1:**
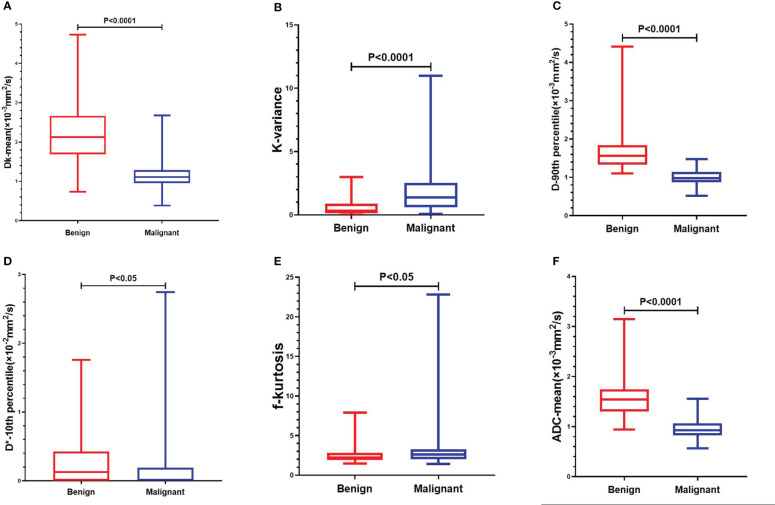
Comparison of whole-lesion histogram parameters of benign and malignant SPL. **(A)**: Dk-mean, **(B)**: K-kariance, **(C)**:D-90th percentile, **(D)**: D*-10th percentile, **(E)**:f-kurtosis, **(F)**: ADC-mean.

**Figure 2:**
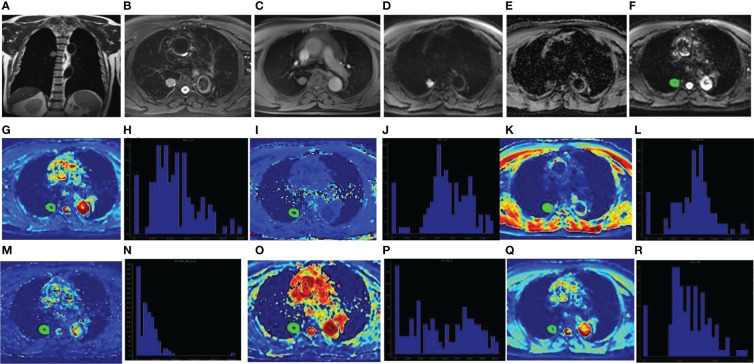
Male, 67 years old, the dorsal segment of right lower lobe showed solid SPN with a length of about 22mm. The pathological findings were adenocarcinoma. Coronal T2WI showed Slightly higher signal **(A)**; Axial T2WI and fat suppression sequence showed uneven slightly higher signal with rough edges and adjacent pleural depression **(B)**; Axial T1WI showed isosignal **(C)**; DWI showed high signal **(D)** and ADC value decreased **(E)**. The ROI was delineated layer by layer on the original image **(F)** in the DKI model, and then a VOI was synthesized and copied to the pseudo-color images of Dk **(G)**, K **(I)**, D **(K)**, D* **(M)**, f **(O)** and ADC **(Q)**. The whole-lesion histogram parameters of Dk **(H)**, K **(J)**, D **(L)**, D* **(N)**, f **(P)** and ADC **(R)** value were calculated. D* is one of the parameters of IVIM.

The interobserver reproducibility ranged from good to excellent for the DKI, IVIM and ADC parameters, and it was finally decided to use the mean of the measurements by two radiologists for statistical analysis. The Dk values of the mean, 10th percentile, 90th percentile, IQR, max, median, min, range, energy, entropy, kurtosis, skewness, and variance of malignant SPLs were significantly lower than those of benign SPLs, while the uniformity was significantly higher than that of benign SPLs. The K values of the 90th percentile, IQR, max, median, min, range and variance of malignant SPLs were significantly higher than those of benign SPLs, while 10th percentile and kurtosis were significantly lower than those of benign SPLs. The D values of the mean, 10th percentile, 90th percentile, IQR, max, median, min, range, energy, entropy and variance of malignant SPLs were significantly lower than those of benign SPLs, while the uniformity was significantly higher than that of benign SPLs. The D* value of the 10th percentile of malignant SPLs was significantly lower than that of benign SPLs. The f value of the kurtosis of malignant SPLs was significantly higher than that of benign SPLs. The ADC value of the mean, 10th percentile, 90th percentile, IQR, max, median, min, range, energy, entropy and variance of malignant SPLs were significantly lower than those of benign SPLs, while the uniformity was significantly higher than that of benign SPLs. All the above differences were statistically significant (P < 0.05) (**Figure 1** and **Supplementary Table S1**). The imaging features and whole-lesion histogram analysis of typical SPLs are shown in **Figure 2**.

### 3.3 Diagnostic efficacy of whole-lesion histogram parameters

For the Dk value, mean had the highest diagnostic efficiency, with an AUC of 0.901, sensitivity of 85.71% and specificity of 91.04%. For the K value, variance had the highest diagnostic efficiency, with an AUC of 0.784, sensitivity of 65.71% and specificity of 82.09%. For the D value, the 90th percentile had the highest diagnostic efficiency, with an AUC of 0.952, sensitivity of 98.57% and specificity of 80.60%. The D* value of the 10th percentile with an AUC of 0.613, sensitivity of 92.86% and specificity of 35.82%. The f value of the kurtosis with an AUC of 0.619, sensitivity of 61.43% and specificity of 67.16%. For the ADC value, the mean had the highest diagnostic efficiency, with an AUC of 0.930, sensitivity of 88.57% and specificity of 85.07%.

Statistically significant single histogram parameters of Dk, K, D and ADC values were combined for statistical analysis. The AUC of the Dk value was 0.951, the sensitivity was 91.43%, and the specificity was 88.06%. The AUC of the K value was 0.834, the sensitivity was 70.00%, and the specificity was 86.57%. The AUC of the D value was 0.967, the sensitivity was 90.00%, and the specificity was 94.03%. The AUC of the ADC value was 0.947, the sensitivity was 94.29%, and the specificity was 82.09%. The AUC of the Dk, D and ADC value were significantly higher than that of the K value (P < 0.05), and there were no significant differences between the other AUCs (P > 0.05).

**Figure 3:**
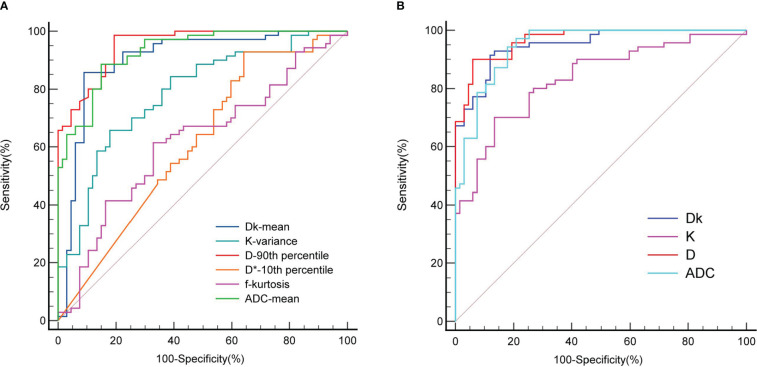
ROC curve for differentiating benign and malignant SPL by whole-lesion histogram parameters. **(A)** is the parameter ROC of single whole-lesion histogram, **(B)** is the multi-parameter ROC of combined whole-lesion histogram. D* is one of the parameters of IVIM.

The diagnostic efficiency of the combined whole-lesion multiparameter histogram was higher than that of a single parameter (**Table 2**, **Figure 3**). By comparing and analyzing the AUC of a single histogram parameter with the highest diagnostic efficiency for Dk, K, D, ADC and the AUC of the combined whole-lesion multiparameter histogram, it was found that the AUC of the Dk value was significantly higher than that of the Dk mean (P = 0.01). There was no significant difference in residual AUC between the two groups (P > 0.05).

**Table 2:** Correlation analysis of whole-lesion histogram parameters with malignant solitary pulmonary lesions.

Parameters		Correlation coefficient (*r_s_*)^a^	Threshold	AUC	Sensitivity (%)	Specificity (%)
Dk (×10^-3^mm^2^/s)	Mean	-0.695	1.389	0.901	85.71	91.04
	10th percentile	-0.542	0.842	0.813	72.86	82.09
	90th percentile	-0.691	1.856	0.899	80.00	91.04
	IQR	-0.455	0.401	0.762	54.29	94.03
	Max	-0.695	2.237	0.901	72.86	94.03
	Median	-0.659	1.356	0.880	84.29	85.07
	Min	-0.388	0.867	0.724	84.29	58.21
	Range	-0.574	1.873	0.831	68.57	83.58
	Energy	-0.662	9.155	0.882	91.43	83.58
	Entropy	-0.486	4.476	0.780	70.00	80.60
	Kurtosis	-0.193	2.487	0.612	44.29	82.09
	Skewness	-0.391	0.603	0.726	77.14	67.16
	Uniformity	0.506	0.510	0.792	65.71	83.58
	Variance	-0.536	1.478	0.809	61.43	91.04
	Dk^b^	–	–	0.951	91.43	88.06
K	10th percentile	-0.321	0.094	0.681	60.00	79.10
	90th percentile	0.423	0.914	0.744	74.29	64.18
	IRQ	0.402	0.456	0.732	62.86	83.58
	Max	0.325	0.862	0.687	94.29	40.30
	Median	0.230	0.749	0.633	60.00	74.63
	Min	-0.257	0.305	0.619	91.43	35.82
	Range	0.377	0.862	0.718	84.29	62.69
	Kurtosis	-0.237	2.053	0.637	48.57	91.04
	Variance	0.491	0.915	0.784	65.71	82.09
	K^b^	–	–	0.834	70.00	86.57
D (×10^-3^mm^2^/s)	Mean	-0.733	1.144	0.923	98.57	71.64
	10th percentile	-0.611	0.862	0.853	91.43	74.63
	90th percentile	-0.783	1.282	0.952	98.57	80.60
	IQR	-0.307	0.242	0.678	82.86	56.72
	Max	-0.753	1.403	0.935	97.14	80.60
	Median	-0.708	1.149	0.909	98.57	68.66
	Min	-0.542	0.742	0.813	87.14	73.13
	Range	-0.331	0.736	0.691	74.29	61.19
	Energy	-0.556	4.675	0.821	91.43	64.18
	Entropy	-0.291	4.112	0.668	81.43	53.73
	Uniformity	0.339	0.586	0.696	91.43	47.76
	Variance	-0.351	0.472	0.703	87.14	49.25
	D^b^	–	–	0.967	90.00	94.03
D* (×10^-2^mm^2^/s)	10th percentile	-0.203	0.335	0.613	92.86	35.82
f	Kurtosis	0.206	2.456	0.619	61.43	67.16
ADC (×10^-3^mm^2^/s)	Mean	-0.744	1.159	0.930	88.57	85.07
	10th percentile	-0.641	0.989	0.870	88.57	73.13
	90th percentile	-0.697	1.469	0.903	81.43	86.57
	IQR	-0.337	0.353	0.695	81.43	53.73
	Max	-0.645	1.588	0.873	74.29	83.58
	Median	-0.727	1.079	0.920	82.86	88.06
	Min	-0.521	0.865	0.801	85.71	70.15
	Range	-0.333	1.678	0.692	95.71	44.78
	Energy	-0.586	6.981	0.838	98.57	68.66
	Entropy	-0.255	4.561	0.647	92.86	43.28
	Uniformity	0.299	0.431	0.673	98.57	34.33
	Variance	-0.342	0.842	0.698	81.43	58.21
	ADC^b^	–	–	0.947	94.29	82.09

Note: ^a^, r_s_ > 0 is positively correlated, while r_s_ < 0 is negatively correlated; ^b^, the combined whole-lesion histogram with multiple parameters; IQR, Inter-quartile range.

In addition, we also conducted a comparative study on the diagnostic efficiency of MRI and CT. The diagnostic accuracy, sensitivity and specificity of MRI and CT were 92.70% (127/137) vs 89.78% (123/137) (p=0.393), 92.86%(65/70) vs 91.43%(64/70) (p=0.753), and 92.54%(62/67) vs 88.06% (59/67) (p=0.381).

### 3.4 Correlation between whole-lesion histogram parameters and malignant SPLs

Most of the histogram parameters for Dk, D and ADC are negatively correlated with malignant SPLs, while most of the histogram parameters for K are positively correlated with malignant SPLs (**Table 2**), indicating that the smaller Dk, D and ADC are and the larger K is, the more likely the SPL is to be malignant. The Dk mean value had the highest correlation with malignant SPLs, with a moderate negative correlation (*r_s_* = -0.695, P < 0.001). The D 90th percentile value had the highest correlation with malignant SPLs, with a highly negative correlation (*r_s_* = -0.783, P < 0.001). The ADC mean value had the highest correlation with malignant SPLs, with a highly negative correlation (*r_s_* = -0.744, P < 0.001). The K variance value had the highest correlation with malignant SPLs, with a moderate positive correlation (*r_s_* =0.491, P < 0.001).

## 4 Discussions

Magnetic resonance imaging is a rapidly developing imaging technology. Due to its high soft tissue resolution; multidirectional, multisequence, multiparameter and multifunctional imaging; few adverse reactions to contrast agents; and lack of ionizing radiation, MRI has been widely used for chest diseases in recent years. MR functional imaging techniques, such as DCE, IVIM and DKI, can not only show the morphological characteristics of the lesions but also obtain the functional information of the internal diffusion and perfusion of the lesions, which can noninvasively evaluate benign vs. malignant lesions, the pathological types of tumors, the degree of differentiation and the efficacy and prognosis of the lesions. It is difficult to find pure ground glass nodules and lung nodules under 10 mm by MRI. It is generally not used for the detection of lung lesions but is mostly used for the diagnostic study of lung nodules and masses. At present, it has not been widely used in clinical practice, and it is still in the research and exploration stage. In this study, the diagnostic accuracy of MRI was higher than that of CT, but the difference was not statistically significant. MRI is not as good as CT in showing the details of pulmonary nodules, but its DWI, IVIM, DKI, DCE and other functional imaging is helpful for the diagnosis and analysis of pulmonary nodules.

The results of this study showed that in the differentiation of benign and malignant SPLs by DKI, IVIM and ADC functional imaging, the mean values of the diffusion-related parameters Dk, D and ADC of malignant SPLs were significantly lower than those of benign SPLs. The results of this study were consistent with those of Wan et al. (18), while Zheng et al. (17) hold that there was no significant difference in the average Dk values of benign and malignant lung lesions. Compared with previous studies on benign and malignant lung lesions by DKI, IVIM and ADC, this study used the whole-lesion histogram analysis method, which can more comprehensively and objectively reflect the complexity and heterogeneity of the microenvironment structure in diseased tissue. DKI (Dk, K), IVIM (D) and ADC can effectively distinguish benign and malignant SPLs. The diagnostic efficiency of the diffusion-related parameters Dk, D and ADC is higher than that of K, D* and f, and the diagnostic efficiency when combined with multiple parameters of the whole-lesion histogram is higher than that of a single parameter. The diagnostic efficiency of combined multiple parameters of the whole-lesion histogram of the D value was the highest, with an AUC of 0.967, specificity of 90.00% and sensitivity of 94.03%.

In DKI whole-lesion histogram analysis, percentile can reflect the distribution of voxels forming the DKI histogram and quantify the heterogeneity of tissue microstructure. Compared with the mean, which is more affected by extreme values, percentile can better reflect small changes in the lesion. Lower percentiles in the Dk, D or ADC histogram (such as the 10th percentile) may represent an area of higher cell density within the lesion. In contrast, higher percentiles (such as the 90th percentile) may represent areas with lower cell density within the lesion and less restricted diffusion of water molecules, such as areas of cystic degeneration, necrosis, and liquefaction (22). Min in the Dk, D or ADC histogram represents the most tightly packed part of the tissue cells, and max represents the part of the lesion with the most free diffusion of water molecules; min and max are at the edge of the histogram, which are susceptible to noise, artifacts, and partial volume effects and have relatively low stability (23). In this study, the 10th percentile, 90th percentile, max and min of Dk, D and ADC of malignant SPLs were significantly lower than those of benign SPLs, which was due to the faster proliferation rate, higher cell density and larger nuclear-cytoplasmic ratio of malignant SPL, resulting in more obvious reduction of extracellular space and more severe diffusion restriction of water molecules. Yuan et al. (24) showed that IVIM-DWI histogram whole-lesion ROI analysis had higher repeatability and diagnostic accuracy than single-level ROI analysis and could provide the biological characteristics and reflect the heterogeneity of the whole tumor. Compared with the ADC value, the D value was more accurate in differentiating benign and malignant SPLs, with the highest accuracy in the D 10th percentile value (81.0%).

In the histogram, energy represents the thickness and uniformity of the gray value distribution, and entropy represents the complexity and nonuniformity of the image’s gray texture. The energy of malignant lesions is lower, and the entropy is larger (25). In this study, the energy of the Dk, D and ADC values of malignant SPLs was significantly lower than that of benign SPLs, which is consistent with the above results. However, the entropy of the Dk, D and ADC values of malignant SPLs was significantly lower than that of benign SPLs, which was inconsistent with the above conclusion, which may be related to the small sample size and small lesion size. The skewness in the DKI histogram reflects the heterogeneity of the voxel distribution, and the larger the absolute value is, the greater the difference in the internal composition of the lesion. Kurtosis reflects the degree to which the distribution of voxels deviates from the Gaussian distribution. When the distribution of voxels is steeper than the Gaussian distribution, the kurtosis value is > 3; conversely, the kurtosis value is < 3 when the skewness value of the Gaussian distribution is 0. The higher the kurtosis value in the Dk, D or ADC histogram, the denser the internal structure of the diseased tissue is, while the larger the skewness value is, the more complex the internal components of the diseased tissue (24). Energy, entropy, kurtosis, and skewness are closely related to the heterogeneity and complexity of tissue microstructure; that is, smaller energy, larger entropy, lower kurtosis, and larger skewness indicate higher heterogeneity and complexity of the diseased tissue (26). In this study, the Dk skewness and kurtosis, K kurtosis values of malignant SPLs were significantly lower than those of benign SPLs, while the kurtosis and skewness values of D and ADC showed no significant difference between benign and malignant SPLs. This may be because the differences in the internal tissue microenvironment structure between benign and malignant SPLs do not lead to significant differences in kurtosis and skewness values.

In this study, most of the histogram parameters for the K value of malignant SPLs were higher than those of benign SPLs, indicating that the diffusion in malignant SPLs was more complex and variable, and the tissue microenvironment structure was more heterogeneous and complex. This is because the growth rate of malignant SPLs is faster, cell proliferation is more active, and the chance of necrosis, hemorrhage and cystic degeneration of tissues and cells is greater, resulting in more obvious internal heterogeneity of lesions. This is consistent with the findings of Zheng (17) and Das SK (27) et al. However, Wan et al. (18) showed that there was no significant difference in the K value between benign and malignant SPLs. The D* value largely depends on the capillary density of the diseased tissue, is sensitive to blood flow velocity and is related to the signal-to-noise ratio, but its repeatability is poor (28). The f value is affected not only by the perfusion characteristics of diseased tissue but also by other factors, such as the relaxation effect, echo time and T2 contribution (8, 29), which may reduce its diagnostic efficacy to some extent. The D* and f values have poor repeatability and large variability and are easily affected by the shape, size, and location of the lesion. In this study, the IVIM-related perfusion parameters D* and f of the whole-lesion histogram parameters, reflecting the microcirculation blood supply of tissue lesions, had little effect on differentiating benign and malignant SPLs, which is consistent with previous findings (17, 18, 29) and may be related to increased capillary angiogenesis not only in malignant lesions but also in benign lesions and the overlap of perfusion between benign and malignant lesions. The conventional IVIM model is prone to produce high amount of noise in estimating perfusion parameters. Therefore, different advanced IVIM models have been proposed in recent years (30–32), which have improved the diagnostic accuracy of IVIM perfusion parameters. However, these advanced models were unfortunately not used in this study. Jiang et al. (33) showed that there was no significant difference in the mean values of D* and f between benign and malignant lung nodules, but the mean values of D* in malignant lung masses were significantly lower than those in benign ones. The value of IVIM-related perfusion parameters needs to be further explored.

Most of the histogram parameters for Dk, D and ADC are negatively correlated with malignant SPLs, while most of the histogram parameters for K are positively correlated with malignant SPLs, reflecting the multiple complex diffusion forms of malignant SPLs, indicating that the internal structure of malignant SPLs is more complex and heterogeneous. The diffusion-related parameters Dk, D and ADC of malignant SPLs were lower than those of benign SPLs because the proliferation rate of malignant SPL cells was faster, the cell density was higher, the extracellular space was smaller, and the diffusion movement restriction of water molecules was more obvious. The K value of malignant SPLs was higher than that of benign SPLs, indicating that the internal microstructure of malignant SPLs was more complex. Zheng et al. (17) conducted logistic regression analysis on 55 cases of benign and malignant lung lesions and found that the regression coefficients of ADC and D were -4.528 and -5.064, respectively, and the regression coefficient of K was 5.606. Other parameters had no statistical significance, indicating that the lower the values of ADC and D and the higher the value of K are, the greater the possibility of the SPL being malignant. Yuan et al. (24) conducted regression analysis on malignant SPLs, and univariate logistic regression analysis showed that the D-value histogram parameters 10th percentile and kurtosis had statistical significance. After adjusting the corresponding D-value histograms by multiple linear regression analysis, kurtosis was not statistically significant, and 10th percentile was an independent predictor for differentiating malignant from benign SPLs.

At present, there is still controversy about the studies of DKI and IVIM on benign and malignant lung lesions. Different researchers (8, 17, 18, 24, 27, 29, 33) have reported different results, which may be related to inconsistencies in individual error, scanning scheme, ROI outline method, postprocessing method, and qualitative and quantitative analysis, which need to be standardized by official guidelines. How to optimize and integrate these functional imaging techniques for clinical application remains to be explored in the future.

There are some limitations in this study. First, this study is a single-center, small-sample study, and only solid or partially solid SPLs > 10 mm were included, so there is a certain selection bias, which may affect the results, and the spatial resolution of MRI should be improved. Second, due to the small number of cases and diseases, regression analysis could not be performed to obtain independent predictors. Larger multicenter, large-sample, and prospective studies are needed to further explore and confirm the clinical application value of DKI and IVIM in lung diseases. Third, there is no unified standard for setting the b value. This study referred to many previous studies and the trade-off between acquisition time and signal-to-noise ratio, which may lead to deviations in results. Further research is needed to develop a more accurate scanning scheme. Fourth, although free breathing acquisition technology was used for scanning, the image quality was somewhat affected by respiratory movement, heartbeat and magnetic sensitivity artifacts, and the MRI examination time was long, so chest MRI scanning technology needs to be further optimized and improved.

## 5 Conclusion

DKI (Dk, K) and IVIM (D) whole-lesion histogram parameters can comprehensively, objectively, and noninvasively reflect the complexity and heterogeneity of the SPL tissue microenvironment structure, and the diagnostic value of the combined multiparameter histogram is higher than that of a single-parameter histogram. The diagnostic efficiency of DKI and IVIM is better than that of the ADC value and can provide additional information about the SPL microstructure, which is helpful in differentiating benign and malignant SPLs and has potential clinical application prospects.

## Data availability statement

The raw data supporting the conclusions of this article will be made available by the authors, without undue reservation.

## Ethics statement

The studies involving human participants were reviewed and approved by Ethics Committee of Chongqing University Three Gorges Hospital. The patients/participants provided their written informed consent to participate in this study. Written informed consent was obtained from the individual(s) for the publication of any potentially identifiable images or data included in this article.

## Author contributions

All authors contributed to the study conception and design. Material preparation, data collection and analysis were performed by LX, HY and XL. The first draft of the manuscript was written by LX and all authors commented on previous versions of the manuscript. All authors read and approved the final manuscript.
