# Supplementary material for: Differential value of diffusion kurtosis imaging and intravoxel incoherent motion in benign and malignant solitary pulmonary lesions
Source: Front Oncol. 2023 Jan 12;12:1075072. doi: 10.3389/fonc.2022.1075072 (PMC9878824; doi:10.3389/fonc.2022.1075072)
Supplement: Supplementary file 1 [file Table_1.docx]

**Supplementary table 1** Comparison of whole-lesion histogram parameters between benign and malignant solitary pulmonary lesions.

| Whole-lesion histogram parameters | | Benign (n=67) | Malignant (n=70) | t/Z value | P value | Adjust P value* |
| --- | --- | --- | --- | --- | --- | --- |
| Dk（×10^-3^㎜^2^/s） | Mean | 2.129 (1.690~2.660) | 1.107 (0.951~1.287) | -8.100 | P＜0.001 | P＜0.004 |
|  | 10th Percentile | 1.394 (0.874~1.750) | 0.704 (0.427~0.894) | -6.318 | P＜0.001 | P＜0.004 |
|  | 90th Percentile | 3.190 (2.093~4.670) | 1.560 (1.364~1.832) | -8.057 | P＜0.001 | P＜0.004 |
|  | IQR | 0.817 (0.630~1.496) | 0.384 (0.263~0.818) | -5.301 | P＜0.001 | P＜0.004 |
|  | Max | 3.660 (2.953~6.021) | 1.834 (1.568~2.426) | -8.104 | P＜0.001 | P＜0.004 |
|  | Median | 1.963 (1.396~2.300) | 1.109 (0.913~1.285) | -7.682 | P＜0.001 | P＜0.004 |
|  | Min | 0.921 (0.447~1.363) | 0.430 (0.065~0.744) | -4.531 | P＜0.001 | P＜0.004 |
|  | Range | 2.976 (2.038~5.167) | 1.334 (0.866~2.288) | -6.692 | P＜0.001 | P＜0.004 |
|  | Energy | 19.908 (12.044~36.475) | 4.487 (3.187~7.027) | -7.717 | P＜0.001 | P＜0.004 |
|  | Entropy | 4.808 ± 0.644 | 4.254 ± 0.488 | 6.233 | P＜0.001 | P＜0.004 |
|  | Kurtosis | 2.930 (2.495~3.873) | 2.614 (2.122~3.254) | -2.256 | 0.024 | 0.041 |
|  | Skewness | 0.809 (0.325~1.077) | 0.197 (-0.175~0.603) | -4.565 | P＜0.001 | P＜0.004 |
|  | Uniformity | 0.385 (0.313~0.487) | 0.579 (0.442~0.747) | -5.902 | P＜0.001 | P＜0.004 |
|  | Variance | 5.833 (2.486~15.577) | 1.089 (0.445~3.988) | -6.248 | P＜0.001 | P＜0.004 |
| K | Mean | 0.582 (0.488~0.842) | 0.734 (0.522~0.834) | -1.809 | 0.071 | 0.119 |
|  | 10th Percentile | 0.382 (0.107~0.520) | 0 (0~0.439) | -3.745 | P＜0.001 | P＜0.004 |
|  | 90th Percentile | 0.841 (0.673~1.064) | 1.110 (0.905~1.277) | -4.928 | P＜0.001 | P＜0.004 |
|  | IQR | 0.239 (0.151~0.335) | 0.565 (0.235~0.850) | -4.687 | P＜0.001 | P＜0.004 |
|  | Max | 1.037 (0.792~1.340) | 1.212 (1.018~1.526) | -3.785 | P＜0.001 | P＜0.004 |
|  | Median | 0.629 (0.510~0.826) | 0.801 (0.557~0.924) | -2.687 | 0.007 | 0.013 |
|  | Min | 0 (0~0.355) | 0 (0~0) | -2.997 | 0.003 | 0.006 |
|  | Range | 0.792 (0.525~1.340) | 1.175 (0.916~1.526) | -4.401 | P＜0.001 | P＜0.004 |
|  | Energy | 1.834 (1.000~2.094) | 1.903 (0.958~3.286) | -1.451 | 0.147 | 0.233 |
|  | Entropy | 3.857 (3.413~4.228) | 3.690 (3.154~4.079) | -0.960 | 0.337 | 0.480 |
|  | Kurtosis | 2.963 (2.200~3.778) | 2.091 (1.657~3.873) | -2.769 | 0.006 | 0.011 |
|  | Skewness | -0.421 (-0.808~0.062) | -0.380 (-1.048~0.162) | -0.099 | 0.921 | 0.967 |
|  | Uniformity | 0.801 (0.657~1.157) | 0.924 (0.693~1.688) | -1.520 | 0.128 | 0.207 |
|  | Variance | 0.319 (0.143~0.892) | 1.368 (0.608~2.523) | -5.732 | P＜0.001 | P＜0.004 |
| D（×10^-3^㎜^2^/s） | Mean | 1.311 (1.017~1.468) | 0.807 (0.705~0.913) | -8.552 | P＜0.001 | P＜0.004 |
|  | 10th Percentile | 1.019 (0.850~1.280) | 0.679 (0.527~0.755) | -7.172 | P＜0.001 | P＜0.004 |
|  | 90th Percentile | 1.561 (1.327~1.833) | 0.981 (0.874~1.139) | -9.134 | P＜0.001 | P＜0.004 |
|  | IQR | 0.288 (0.114~0.405) | 0.156 (0.109~0.225) | -3.585 | P＜0.001 | P＜0.004 |
|  | Max | 1.745 (1.412~2.019) | 1.112 (0.974~1.245) | -8.780 | P＜0.001 | P＜0.004 |
|  | Median | 1.313 (0.984~1.472) | 0.801 (0.676~0.900) | -8.259 | P＜0.001 | P＜0.004 |
|  | Min | 0.932 (0.676~1.168) | 0.586 (0.396~0.693) | -6.322 | P＜0.001 | P＜0.004 |
|  | Range | 0.853 (0.461~1.115) | 0.537 (0.338~0.757) | -3.856 | P＜0.001 | P＜0.004 |
|  | Energy | 5.936 (3.192~9.417) | 2.422 (1.650~3.628) | -6.481 | P＜0.001 | P＜0.004 |
|  | Entropy | 4.143 (3.432~4.440) | 3.677 (3.375~4.006) | -3.389 | 0.001 | 0.002 |
|  | Kurtosis | 2.639 (2.224~3.360) | 2.767 (2.174~3.383) | -0.629 | 0.530 | 0.685 |
|  | Skewness | 0.216 ± 0.644 | 0.160 ± 0.671 | 0.495 | 0.621 | 0.767 |
|  | Uniformity | 0.625 (0.509~1.031) | 0.875 (0.716~1.161) | -3.953 | P＜0.001 | P＜0.004 |
|  | Variance | 0.433 (0.126~0.833) | 0.167 (0.067~0.336) | -4.095 | P＜0.001 | P＜0.004 |
| D*（×10^-2^㎜^2^/s） | Mean | 0.728 (0.544~1.077) | 0.706 (0.492~1.002) | -1.270 | 0.204 | 0.295 |
|  | 10th Percentile | 0.128 (0~0.426) | 0.004 (0~0.192) | -2.367 | 0.018 | 0.032 |
|  | 90th Percentile | 1.384 (1.030~1.748) | 1.248 (0.928~1.927) | -1.339 | 0.180 | 0.272 |
|  | IQR | 0.616 (0.439~0.781) | 0.620 (0.464~0.851) | -0.472 | 0.637 | 0.764 |
|  | Max | 2.216 (1.500~3.394) | 2.129 (1.366~3.770) | -0.198 | 0.843 | 0.920 |
|  | Median | 0.698 (0.496~0.854) | 0.580 (0.356~0.886) | -1.636 | 0.102 | 0.168 |
|  | Min | 0 (0~0) | 0 (0~0) | -0.149 | 0.882 | 0.950 |
|  | Range | 2.212 (1.500~3.394) | 2.068 (1.362~3.759) | -0.357 | 0.721 | 0.841 |
|  | Energy | 3.186 (1.473~7.419) | 3.219 (1.332~8.158) | -0.280 | 0.780 | 0.885 |
|  | Entropy | 4.379 (3.907~4.869) | 4.337 (3.802~4.720) | -1.339 | 0.180 | 0.272 |
|  | Kurtosis | 3.615 (2.767~5.258) | 3.567 (2.427~7.157) | -0.706 | 0.480 | 0.640 |
|  | Skewness | 0.750 (0.416~1.229) | 0.980 (0.282~2.072) | -1.292 | 0.196 | 0.289 |
|  | Uniformity | 0.571 (0.385~1.003) | 0.649 (0.440~1.072) | -1.361 | 0.174 | 0.271 |
|  | Variance | 2.351 (1.667~4.024) | 2.621 (1.159~8.601) | -0.047 | 0.962 | 0.998 |
| **f** | Mean | 0.215 (0.143~0.277) | 0.187 (0.109~0.281) | -0.891 | 0.373 | 0.522 |
|  | 10th Percentile | 0.004 (0~0.125) | 0.006 (0~0.086) | -0.233 | 0.816 | 0.902 |
|  | 90th Percentile | 0.402 (0.226~0.572) | 0.345 (0.223~0.520) | -0.661 | 0.509 | 0.668 |
|  | IQR | 0.189 (0.087~0.296) | 0.147 (0.094~0.246) | -0.538 | 0.590 | 0.740 |
|  | Max | 0.492 (0.321~0.621) | 0.443 (0.303~0.636) | -0.484 | 0.628 | 0.765 |
|  | Median | 0.175 (0.101~0.293) | 0.173 (0.093~0.270) | -0.594 | 0.552 | 0.703 |
|  | Min | 0 (0~0.031) | 0 (0~0.015) | -0.760 | 0.447 | 0.606 |
|  | Range | 0.457 (0.280~0.583) | 0.419 (0.293~0.570) | -0.028 | 0.978 | 1.000 |
|  | Energy | 0.236 (0.058~4.182) | 0.177 (0.092~0.386) | -0.788 | 0.431 | 0.594 |
|  | Entropy | 3.396 (2.709~3.711) | 3.368 (2.940~3.647) | -0.276 | 0.783 | 0.877 |
|  | Kurtosis | 2.198 (1.908~2.830) | 2.629 (2.028~3.266) | -2.407 | 0.016 | 0.029 |
|  | Skewness | 0.226 (-0.226~0.636) | 0.205 (-0.191~0.823) | -0.017 | 0.986 | 0.998 |
|  | Uniformity | 1.111 (0.941~1.739) | 1.197 (0.961~1.628) | -0.366 | 0.714 | 0.845 |
|  | Variance | 0.143 (0.042~0.308) | 0.110 (0.058~0.289) | -0.353 | 0.724 | 0.833 |
| ADC（×10^-3^㎜^2^/s） | Mean | 1.540 (1.299~1.740) | 0.928 (0.826~1.062) | -8.677 | P＜0.001 | P＜0.004 |
|  | 10th Percentile | 1.151 (0.901~1.397) | 0.745 (0.609~0.897) | -7.476 | P＜0.001 | P＜0.004 |
|  | 90th Percentile | 1.928 (1.565~2.681) | 1.128 (1.019~1.411) | -8.130 | P＜0.001 | P＜0.004 |
|  | IQR | 0.353 (0.181~0.552) | 0.205 (0.123~0.334) | -3.932 | P＜0.001 | P＜0.004 |
|  | Max | 2.416 (1.594~3.177) | 1.241 (1.137~1.602) | -7.525 | P＜0.001 | P＜0.004 |
|  | Median | 1.488 (1.263~1.697) | 0.926 (0.799~1.056) | -8.477 | P＜0.001 | P＜0.004 |
|  | Min | 1.008 (0.735~1.372) | 0.635 (0.500~0.791) | -6.079 | P＜0.001 | P＜0.004 |
|  | Range | 1.220 (0.569~1.959) | 0.619 (0.399~1.050) | -3.880 | P＜0.001 | P＜0.004 |
|  | Energy | 10.300 (4.279~14.224) | 3.449 (2.179~4.867) | -6.830 | P＜0.001 | P＜0.004 |
|  | Entropy | 3.948（3.655~4.739） | 3.878 (3.515~4.291) | -2.976 | 0.003 | 0.006 |
|  | Kurtosis | 2.922 (2.200~3.631) | 2.790 (2.273~3.526) | -0.116 | 0.907 | 0.964 |
|  | Skewness | 0.483 (0.029~0.716) | 0.498 (-0.003~0.822) | -0.009 | 0.993 | 0.993 |
|  | Uniformity | 0.679 (0.416~0.865) | 0.820 (0.565~1.025) | -3.488 | P＜0.001 | P＜0.004 |
|  | Variance | 0.932 (0.174~3.091) | 0.256 (0.106~0.733) | -3.992 | P＜0.001 | P＜0.004 |

Note: two independent sample t-test were expressed as mean ± standard deviation; Mann-Whitney U test were expressed as M (P25, P75); IQR,Inter-quartile Range. * The FDR-corrected P-values were used.
